# Influence of the Architecture of Soft Polymer-Functionalized Polymer Nanoparticles on Their Dynamics in Suspension

**DOI:** 10.3390/polym12081844

**Published:** 2020-08-17

**Authors:** Young-Gon Kim, Waraporn Wichaita, Héloïse Thérien-Aubin

**Affiliations:** Max Planck Institute for Polymer Research, Ackermannweg 10, 55131 Mainz, Germany; kimyoung@mpip-mainz.mpg.de (Y.-G.K.); waraporn.wic@gmail.com (W.W.)

**Keywords:** soft nanoparticles, grafted polymer chains, grafting density, NMR, relaxation

## Abstract

The behavior of nanogels in suspension can be dramatically affected by the grafting of a canopy of end-tethered polymer chains. The architecture of the interfacial layer, defined by the grafting density and length of the polymer chains, is a crucial parameter in defining the conformation and influencing the dynamics of the grafted chains. However, the influence of this architecture when the core substrate is itself soft and mobile is complex; the dynamics of the core influences the dynamics of the tethered chains, and, conversely, the dynamics of the tethered chains can influence the dynamics of the core. Here, poly(styrene) (PS) particles were functionalized with poly(methyl acrylate) (PMA) chains and swollen in a common solvent. NMR relaxation reveals that the confinement influences the mobility of the grafted chain more prominently for densely grafted short chains. The correlation time associated with the relaxation of the PMA increased by more than 20% when the grafting density increased for short chains, but for less than 10% for long chains. This phenomenon is likely due to the steric hindrance created by the close proximity to the rigid core and of the neighboring chains. More interestingly, a thick layer of a densely grafted PMA canopy efficiently increases the local mobility of the PS cores, with a reduction of the correlation time of more than 30%. These results suggest an interplay between the dynamics of the core and the dynamics of the canopy.

## 1. Introduction

Nanoparticles (NP) possess a large surface area to volume ratio, and the control of the interface has a critical influence on the properties of the NP systems by tuning their interaction with their surrounding environment. Controlling the interfacial properties is critical since the incompatibility of the NPs with their surrounding media can result in the agglomeration of the NPs. The interfacial properties of the NPs can be controlled by tethering polymer chains at the interface, to improve the NPs compatibility with a solvent or a polymer matrix [1,2,3,4,5]. The layer of tethered polymers, whether the chains are in a brush conformation or not, forms a canopy around the core particle, and this layer of grafted chains defines the interaction between the NPs and between the NPs and their environment [6]. In such cases, the final behavior of the NPs is influenced by the chemical composition of the grafted chains, but also by their degree of polymerization (*N*) and their grafting density, the number of grafted chains per unit of area (σ). Variations in *N* and σ in systems where the polymer chains are tethered to hard and rigid nanoparticle core, such as silica or gold, influence the dynamics of the grafted polymer chains.And the architecture of the canopy of grafted polymer chains defines the interfacial properties of the NPs, such as particle/particles interaction or NPs/solvent interaction [6,7,8,9,10,11]. However, the behavior of systems where the core is itself soft and deformable like a nanogel has not been addressed, and the dynamics of polymer chains grafted on the surface of soft NPs need to be thoroughly understood to improve the design of such systems [12].

The architecture of the grafted polymer system, defined by the σ and *N* of the canopy, affects the conformation [13,14,15] and the dynamics of the grafted chains [16]. Both of which, in turns, largely affect the macroscopic behavior of systems based on polymer-functionalized NPs, such as the mechanical properties of nanocomposites [16,17] or the rheology of colloidal suspensions [11,16,18]. In a polymer-grafted NPs system, usually, the polymer chains experience a deceleration of the relaxation in comparison to free chains as a consequence of the stretched chain conformation near the surface of nanoparticles and increased confinement generated by the proximity of adjacent chains [19] at least at the scale of the local segmental motion. On a larger scale, the increased confinement experienced by densely grafting chains usually resulted in an increase of glass transition temperature of tethered chains in comparison to the free chains ascribed to the presence of a reduced number of chain ends and smaller free volume in the grafted system [20,21,22].

The investigations of the behavior of polymer grafted nanoparticulate systems have mostly dealt with rigid core NPs like silica or gold. However, when soft and swollen polymer NPs are used as the substrate, the intrinsic dynamics of the polymer chains in the core also needs to be taken into account. In such swollen polymer nanoparticles, the dynamics of the chains is typically constrained and sparsely crosslinked nanogel displayed a soft interface with polymer chains having a rapid relaxation, while densely crosslinked nanogels exhibited glassy surface with constrained local mobility [23,24,25]. In nanocolloids resulting from the self-assembly of block copolymers, this arrested core is surrounded by a canopy of swollen and mobile polymer chains. In such a case, the polymer chains within the core experienced a soft confinement, where the dynamics is influenced by the fluctuation of the interface where the two polymer components are connected [26]. Such phenomena affect the design of soft particles for different applications, for example, theranostic systems [23,25,27]. While block copolymer micelles are an interesting point of comparison to the polymer-functionalized nanoparticles, in such systems, the effect of the density of the surface functionalization on the local dynamics cannot be addressed, since it is difficult to control the packing density.

**Figure 1:**
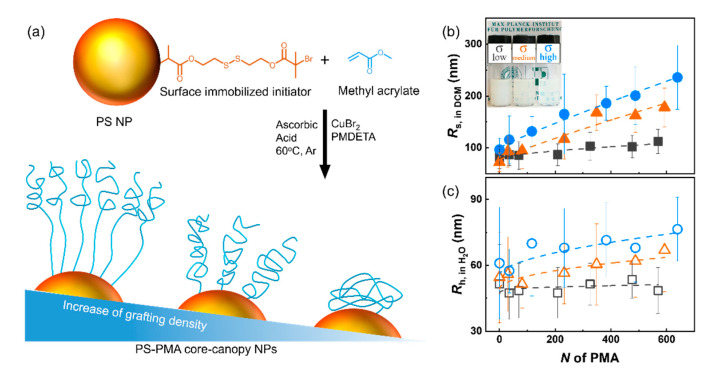
(**a**) Synthesis of poly(styrene) (PS)-poly(methyl acrylate) (PMA) core-canopy nanoparticles (NPs); (**b**) Solvodynamic radii (*R*_s_) and (**c**) hydrodynamic radii (*R*_h_) of PS-PMA NPs in DCM and H_2_O, respectively. For NPs functionalized with PMA chains with a degree of polymerization (*N*) ranging from 0 to 639 units with low (0.17 chains nm^−2^, □), medium (0.8 chains nm^−2^, △) and high (2.5 chains nm^−2^, ○) grafting density, measured by dynamic light scattering at 25 °C. The inset photo in (**b**) displays the suspensions of PS-PMA_49k_-σ_low_, PS-PMA_51k_-σ_medium_, and PS-PMA_55k_-σ_high_ in DCM at a concentration of 16.67 mg mL^−1^.

Here, the combined effects of the *N* and the σ of the grafted canopy on the dynamics of both the core and the canopy of polymer-functionalized polymer nanoparticles were investigated using NMR relaxometry. Polystyrene (PS) NPs functionalized with a canopy of end-grafted poly(methyl acrylate) (PMA) were prepared by a combination of miniemulsion polymerization and surface-initiated polymerization, and dispersed in a common good solvent for both the core and the canopy. The σ was controlled and resulted in different canopies composed of either stretched polymer chains or collapsed chains (Figure 1a). The *N* of the grafted chains was also modified, so the contour length of the grafted PMA chains was increased from ca. 7 to 150 nm, while the radius of the swollen PS core was kept constant at ca. 100 nm. NMR relaxometry gave access to the correlation time (*τ*_c_) associated with the subsegmental dynamics of the polymers [28,29]. The activation energy associated with the subsegmental dynamics of the polymer NPs in suspensions was analyzed and compared to the thermal behavior of the dried NPs.

## 2. Materials and Methods

### 2.1. Materials

The ATRP inimer (2-((2-(3-methyl-2-oxobut-3-en-1yl)xy)ethyl)disulfanyl)ethyl 2-bromo-2-methylpropanoate) was synthesized as previously reported [18,30]. Styrene (99%, Sigma-Aldrich, Darmstadt, Germany), methylacrylate (99%, Sigma-Aldrich, Darmstadt, Germany) and divinylbenzene (80%, Alfa Aesar, Ward Hill, MA, USA) were purified with a basic aluminum oxide column (Sigma-Aldrich, Darmstadt, Germany). 2,2’-azobis(2-methylbutyronitrile) (V-59, WAKO, Osaka, Japan) was recrystallized in methanol (MeOH). All other chemical reagents were used as received.

### 2.2. Synthesis of Nanoparticles Functionalized with ATRP Inimer (PS-Br NPs)

In a typical procedure, 28 mmol of styrene (St, 0.99 eq.), 287 μmol of divinylbenzene (DVB, 0.01 eq.) and 38 mmol of hexadecane (HD, 0.04 eq.) were added to a 40 mL vial and were mixed with 144 μmol of a V-59 (0.005 eq.). After 10 min of mild stirring, 24 mL of 10 mM of aqueous solution of sodium dodecyl sulfate (SDS) was added, and vigorously stirred for 15 min. The mixture was emulsified by using a probe sonicator (Branson 450 Digital Sonifier, Danbury, CT, US) equipped with a titanium solid extender tip, diameter of 1/2” for 2 min at 0 °C (20 kHz, 70% amplitude, 10 s on/2 s off). The resulting miniemulsion was transferred to a 50 mL round bottom flask and heated at 80 °C to initiate polymerization. After 2.5 h of reaction, 2 mL of SDS aqueous solution (250 mM) was added and the reaction mixture was degassed by bubbling with Ar for 10 min. Then, in order to cover PS core NPs with a thin layer of ATRP inimer (Figure 1a), a mixture of St (5.8 mmol, 0.99 eq.), DVB (58 μmol, 0.01 eq.), ATRP inimer (59–590 μmol, 0.01–0.1 eq.) and V-59 (30 μmol) was added dropwise, at a rate of 1 mL h^−1^. The mixture reacted overnight at 80 °C and was then filtered. The PS NPs in the aqueous suspension were purified by precipitation in 200 mL of MeOH and air-dried. To obtain impurity-free PS NPs without HD and SDS, the NPs were redispersed in 40 mL of THF, precipitated in 500 mL of MeOH three times and redispersed in THF and precipitated in hexane. Finally, the NPs were air-dried.

### 2.3. Synthesis of the End-Tethered Canopy of PMA on the Surface of the PS Core (PS-PMA NPs)

The end-tethered PMA canopy was grafted from PS core by surface-initiated ATRP (Figure 1). A solution of methyl acrylate (4.4 mmol) dissolved in 0.1 mL of DMF containing Cu(II)Br_2_ (2000 ppm) and PMDETA (Cu(II):PMDETA = 1:10) was added into a 25 mL Schlenk tube containing a suspension of the PS-Br NPs functionalized with the ATRP initiator (50 mg) dispersed in anisole (4 mL). PDMS (0.1 mL) was added to the suspension as internal standard. The mixture was stirred and degassed with argon for 30 min. In order to initiate the polymerization, 0.5 mL of DMF containing ascorbic acid (1600 ppm) was added. This resulting suspension was further degassed with argon for 10 min, and the reaction vessel was heated at 60 °C and allowed to react. When the targeted monomer conversion was obtained, the reaction mixture was diluted with 10 mL of THF, precipitated in 100 mL of MeOH. The PS-PMA core-canopy NPs were purified three times by redispersing the NPs in 20 mL of DCM and precipitating in 100 mL of *n*-hexane. The NPs were then dried overnight under vacuum. The reaction was repeated by varying the amounts of the monomer, Cu(II)/ligand and bromoisobutyrate grafted moieties. For the free PMA chains, the appropriate molar amount of ethyl α-bromoisobutyrate (Sigma-Aldrich, Darmstadt, Germany) was used as the ATRP initiator in the reaction mixture instead of PS-Br NPs.

### 2.4. Characterization

The solvodynamic radius of the NPs was determined by dynamic light scattering (DLS) measured with a Malvern Instruments Zetasizer Nano S90 (Malvern, UK) at a fixed angle of 90° (λ = 633 nm, 15 runs, run duration of 10 s). The nanoparticles were dispersed either in DCM or in an aqueous solution of CTAC at a concentration of 0.05 mg mL⁻^1^. All the measurements were carried out at 25 °C. The σ was measured by inductively coupled plasma atomic emission spectrometry (ICP-AES) with an ACTIVA M spectrometer (Horiba Jobin Yvon) equipped with a Meinhardt-type nebulizer and a cyclone chamber, and processed by the software ACTIVAnalyst 5.4. The glass transition temperatures of the NPs were measured by using differential scanning calorimetry (DSC, 204F1/ASC Phönix, Netzsch, Selb, Germany) under nitrogen flow of 20 mL min^−1^ between −80 to 200 °C at heating and cooling rates of 10 K min^−1^. The relaxation experiments were performed on a series of NMR AVANCE spectrometers (Bruker, Ettlingen, Germany) working at a nominal frequency of 500.13, 700.02 and 850.27 MHz. The details of NMR experiments are described in Figures S1 and S2, Supplementary Material.

## 3. Results and Discussion

Using SI-ATRP, the PMA chains were successfully grafted from the surface of initiator immobilized PS NPs prepared by miniemulsion, and resulted in the formation of PS NPs having PMA canopies with different σ and varying *N* (Table 1). The increase in size created by the grafting of the PMA canopy resulted in a larger solvodynamic radius (*R*_s_) for the samples with longer PMA chains or higher σ (Figure 1b); similar results were also observed in the dry state by TEM measurements (Figure S3, Supplementary Material).

The comparison of the size of the NPs in DCM and in H_2_O by DLS (Figure 1b,c) made it possible to study the swelling of the NPs and the evolution of thickness of the layer of end-grafted polymer chains. The size of the NPs in both DCM and water increased with increasing *N* and σ. In addition, DCM is a good solvent for both the PS core and the PMA canopy and both components were swollen in suspension, while water is a poor solvent for both the PS and PMA, and the NPs were in a collapsed state. This variable swelling of the core had a consequence on the apparent σ of the system. In water, because the NP cores were collapsed, the number of chains per unit of surface area was larger than for the same NP in DCM (Table 2).

In DCM, *R*_s_ of bare PS NPs with σ_low_ was 81 nm and the size increased up to 110 nm for NPs functionalized with PMA chains with *N* = 569 units. For NPs with σ_high_, *R*_s_ of bare PS NPs was 90 nm and increased up to 240 nm when PMA chains with *N* = 639 units were grafted. The variation of the size of the swollen NPs with the increase of *N* (Figure 1b,c) follows the scaling relation:

*R*_s_ − *R*_s,0_ ∝ *N^v^* (1)

where *R*_s_ is the thickness of grafted chains, *N* is the degree of polymerization, *v* is the scaling exponent and *R*_s,0_ is the size of the PS core. The scaling factor was shown to change for different grafting regime [14,15,31,32]. Here, at σ_low_ in DCM the scaling factor (*v* = 0.63) was typical of grafted chains in the semi-collapsed chain regime in a good solvent. For the NPs with σ_medium_ and σ_high_, the scaling exponent of 0.88 and 0.91 were observed, respectively (Table 2), typical of grafted chains in the stretched regime [14,15]. In water, a poor solvent for the PMA canopy, the stretching factor of the canopy with a σ_low_ was 0.27 typical of collapsed chains. The particles prepared at higher σ_medium_ and σ_high_ dispersed in water showed a stretching parameter corresponding to the semi-collapsed regime. The result obtained for the stretching parameter of the grafted chains the PS-PMA NPs was in keeping with the decrease in solvent quality going from DCM to water. In a good solvent, as a consequence of the polymer-solvent enthalpic attraction and the polymer-polymer entropic repulsion, the grafted polymer chains adopt a stretched and extended conformation [33,34] resulting in a large *v*. Conversely, in a poor solvent, the favorable polymer-polymer attraction results in the adoption of a collapsed conformation. If the NPs were dispersed in a good solvent at a high enough concentration, the formation of colloidal gels was observed [18]. However, the NMR experiments were performed below the solution-to-gel transition to study the dynamics of single particles rather than the dynamics of the NPs network.

The NMR relaxation of the NPs suspension was measured at different magnetic fields and different temperatures to understand the local subsegmental chain dynamics in both the PS core and the PMA canopy. Both the spin-lattice relaxation (*T*_1_) and spin-spin relaxation (*T*_2_) were measured. The relaxation times obtained by NMR spectroscopy are influence by *τ*_c_ of the relaxation process and the interactions between the spin under study and their environment. Typically, *T*_2_ is not largely influenced by the magnetic field used and the values of *T*_2_ can be directly correlated to the subsegmental relaxation [35]. As the local mobility of the molecules under investigation decreases, the value of the *T*_2_ time decreases, and for very slow systems, it can be difficult to quantify the *T*_2_ relaxation precisely without using alternative pulse sequences. Consequently, the measurement of the *T*_2_ of the crosslinked PS core was challenging. Thus, *T*_1_ relaxation time, although providing less precise measurement of the local subsegmental chain relaxation, was also use to probe the PS-PMA NPs.

**Figure 2:**
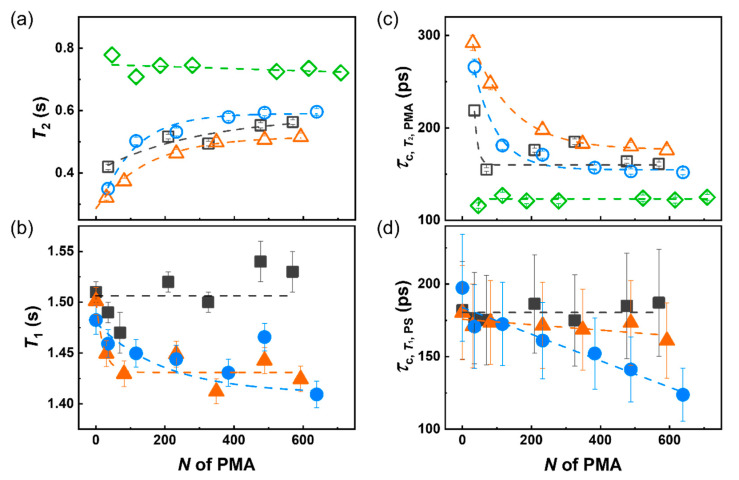
(**a**) ^1^H spin–spin relaxation time of methoxy group of the PMA canopy and (**b**) ^1^H spin-lattice relaxation time of aromatic ring of the PS core in the PS–PMA NPs with different degrees of polymerization of the PMA chains (*N*) grafted on PS cores with different grafting density, measured at 298 K by using an NMR spectrometer at a Larmor frequency of 700.02 MHz; (**c**) Correlation time of ^1^H of methoxy group of PMA canopy obtained from *T*_2_ relaxation and (**d**) correlation time of ^1^H of the aromatic ring of PS core obtained from *T*_1_ relaxation, calculated from BPP theory. For PS-PMA NPs with low (0.17 chains nm^−2^, □), medium (0.8 chains nm^−2^, △), high (2.5 chains nm^−2^, ○) grafting density and free PMA chain (◇).

The *T*_2_ of PMA was used to probe the relaxation dynamics of the tethered chains (Figure 2a). The *T*_2_ of free PMA chains depicted ca. 0.77 s at *N* = 46 and slightly decreased with an increase of *N*. The short tethered PMA chains (*N* = 35) at σ_low_ showed of *T*_2_ of 0.42 s and as chain length increased, the *T*_2_ value also moderately increased. The increase in the *T*_2_ value indicates an enhanced subsegmental mobility of the chains. Thus, it can be concluded that the local mobility of the tethered PMA chains with σ_low_ increased when the *N* of the chain increased. Even though the PMA chains on the PS-PMA NPs at σ_low_ displayed a collapsed brush conformation, given their chain stretching factor (Table 2), their local subsegmental mobility was reduced in comparison to untethered PMA chains of the same length, likely because the presence of the PS substrate restricted the subsegmental motion of the chains. As σ increased from low to high, the *T*_2_ of the short tethered PMA decreased due to the transition from a collapsed conformation to a stretched brush regime (Table 2) generated by the crowding produced by the adjacent chains. The relaxation of the free and untethered PMA chains was not influenced by the *N* in the range of molecular weights studied. However, for the grafted chains, as the chain length increased, the *T*_2_ of the grafted PMA chains systematically increased for all σ. The increase in the local mobility of the grafted PMA chains as *N* increased was attributed to the reduction in the spatial crowding observed as the distance from the core increased with increasing *N* [13]. These observations are in keeping with what has been observed for the local subsegmental dynamics of polymer chains grafted to the rigid nanoparticles [19,36].

In the PS-PMA NPs system, both the PMA and the PS were undergoing relaxation. Addressing the effect of the grafting of polymer chains on the dynamics of the soft core can be the key to explain the divergences observed when comparing the macroscopic properties of soft and hard polymer-functionalized particles [18]. However, the local dynamics of the crosslinked PS core was too slow to be probed efficiently by the measurement of the *T*_2_ (Figure S4, Supplementary Material). In the slow regime, the *T*_1_ of a proton will increase as the local chain *τ*_c_ decreases. Furthermore, the *T*_1_ values are significantly influenced by the local magnetic field. Consequently, to get a clear picture of the chain relaxation in the PS cores *T*_1_ relaxation times were measured at different magnetic fields (Figure S5, Supplementary Material).

The relaxation of all the unfunctionalized PS cores exhibited similar *T*_1_ relaxation times. The grafting of PMA chains of different *N* at σ_low_ resulted in no significant changes in the *T*_1_ value observed. However, when PS cores were functionalized with PMA chains at σ_medium_ and σ_high_, the *T*_1_ of the PS core decreased with the increase of *N*_PMA_. (Figure 2b) Consequently, at σ_medium_ and σ_high_ the grafting of the PMA chains increased the mobility of the PS core, and this effect was more pronounced with longer chains.

The relaxation time *T*_1_ and *T*_2_ obtained by NMR spectroscopy are measures of the relaxation of the spins system, an indirect measurement of the local dynamics, those values can be used to calculate the *τ*_c_ of subsegmental motions of the chains [35]. The average *τ*_c_ of the PMA chains and of the PS core were independently measured from the *T*_2_ for the PMA and *T*_1_ for the PS. The *τ*_c_ of free PMA chains was not significantly affected by *N* (Figure 2c) in the range of molecular weight studied. The grafting of the PMA chains to the PS core resulted in a decrease of in the local subsegmental mobility of the PMA chains resulting in larger *τ*_c_ values for the grafted PMA chains in comparison to the free PMA chains. As the σ increased, the constrain on each chain created by the neighboring chains increased and led to the increase in *τ*_c_ observed especially at low *N*. At every σ as *N* increased, the difference in *τ*_c_ of the free and grafted PMA chains decreased. The *τ*_c_ measured by NMR relaxation is the average *τ*_c_ for the chains, and the decrease in the average *τ*_c_ of the PMA chain with increasing *N* suggested that the reduced dynamics experienced by the PMA segments was more important close to the interface with the PS core, in keeping with the decrease of monomer concentration as the distance from the surface increases, which reduced the crowding generated by neighboring chains [13]. Furthermore, as the length of the chains increased the difference observed for the nanoparticles with different σ decreased, because the segments of the grafted chains farther away from the core are less constrained at every σ, in keeping with observation of polymer chains grafted to hard nanoparticle cores [16].

Furthermore, the grafting of the PMA chains to the PS core influenced the local dynamics of the PS cores. After the grafting of the PMA chain at the interface of the PS NPs, a limited decrease in the *τ*_c_ of the PS core was observed, and this effect was stronger when the surface of the PS NPs was functionalized with long PMA chains (Figure 2d). Furthermore, as σ increased, the impact of the PMA chains on the dynamics of the PS core increased. This result can be attributed to an interplay between the slow dynamics of PS core and faster dynamics of PMA canopy. A similar behavior can be observed for block copolymer when a component with a high relaxation rate, increases the local dynamics of a slower block, because of the increased local fluctuations of the junction between the two segments initiated by the faster component [37,38]. Additionally, the effect of σ suggests that the number of grafted chains, rather than the dynamics of the grafted chains themselves, was the driving force for the faster dynamics observed with the PS core functionalized with a high number of long PMA chains.

**Figure 3:**
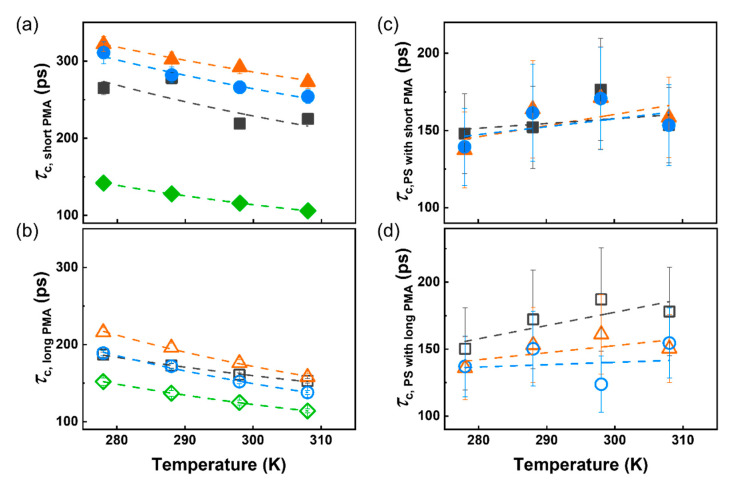
Temperature dependence of correlation time of PMA canopy with (**a**) short chains (*N <* 50), (**b**) long chains (*N* > 500), PS core with (**c**) short PMA chains and (**d**) long PMA chains. For PS-PMA NPs with low (0.17 chains nm^−2^, □), medium (0.8 chains nm^−2^, △), high (2.5 chains nm^−2^, ○) grafting density and free PMA chain (◇).

**Figure 4:**
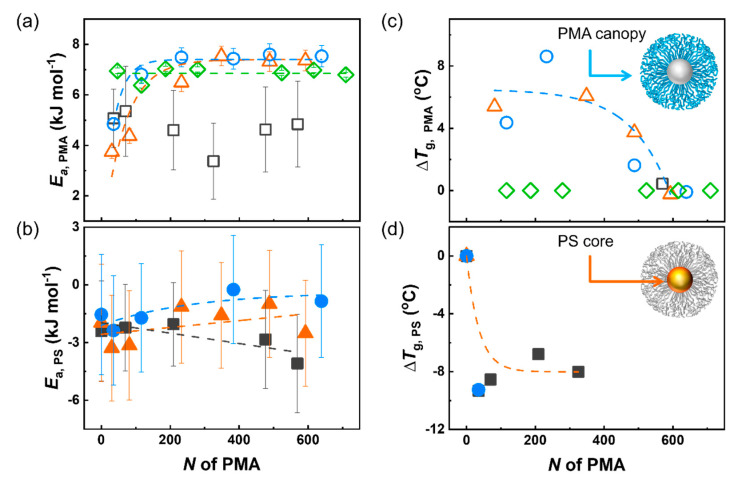
Energy of activation of local segmental motion of (**a**) PMA canopy and (**b**) PS core in PS-PMA NPs; Glass transition temperature (*T*_g_) difference between (**c**) free PMA and tethered PMA in PS-PMA with different degrees of polymerization of the PMA chains (Δ*T*_g,PMA_ = *T*_g,PMA in PS-PMA_ − *T*_g,free PMA_) and (**d**) are PS NPs and PS core of PS-PMA NPs (Δ*T*_g,PS_ = *T*_g,PS in PS-PMA_ − *T*_g,bare PS_). For PS-PMA NPs with low (0.17 chains nm^−2^, □), medium (0.8 chains nm^−2^, △), high (2.5 chains nm^−2^, ○) grafting density and free PMA chain (◇).

The variation of the *τ*_c_ of a solvated polymer at different temperatures (Figure 3) can be described as an Arrhenius relation, where the activation energy associated with the subsegmental relaxation of the chains (Figure 4a,b) is a measure of the cooperativity of the segmental movement [39,40,41]. As the temperature increased, the *τ*_c_ of the PMA chains decreased as expected (Figure 3a,b), since higher temperatures increase the local subsegmental mobility of the polymer. However, in the case of the PS core, the *τ*_c_ increased with an increase in the temperature (Figure 3c,d). This unexpected result is related to the deswelling of crosslinked PS observed at high temperatures in specific solvent systems (Figure S6 Supplementary Material, ) [12,42].

The energy of activation of the PMA chains of PS-PMA core-canopy NPs was calculated using the *τ*_c_ measured at different temperatures (Figure 4a). As expected, the activation energy associated with the subsegmental dynamics of the untethered PMA chains did not change significantly with *N*. However, short grafted chains displayed lower activation energy than the free chains, indicative of a decrease in the degree of cooperativity associated with the relaxation process, in keeping with the conformational changes of the PMA chains upon grafting when the chains adopted a partially stretched conformation. As the *N* increases, the difference in the extent of cooperativity of the relaxation process between the grafted chain and the free chains decreased. As a result of the limited weight fraction of PMA in the PS-PMA samples prepared at a low σ, the measurements were not as precise, and no clear conclusion to the effect of the σ can be drawn. In the case of the PS core, the results are more complex; the deswelling of the PS core led to the reduction in the local subsegmental mobility of the PS core and resulted in apparent negative activation energy for the relaxation process (Figure S4, Supplementary Material). Nonetheless, the results clearly show that the grafting of the PMA chains facilitated the relaxation of the PS core, and this effect was more pronounced for long chains grafted with σ_high_ and σ_medium_ than with σ_low_.

The activation energy measured for the DCM suspensions of the PS-PMA NPs clearly demonstrated an interaction between the dynamics of the PS core and the dynamics of the PMA canopy. Similar trends are also observed in the solid-state. The thermal behavior of dried PS-PMA (Figure 4c,d) showed that both the glass transition temperature of the PS core and of the PMA canopy was affected by the architecture of the PMA canopy. The untethered PMA chains displayed a moderate increase (ca. 2 °C) of glass transition temperature with the *N* as expected from the Flory-Fox equation [43,44]. Short PMA chains grafted to the PS core displayed a glass transition temperature systematically higher than free PMA chains of the same molecular weight. The difference in the glass transition temperature of free and tethered chains decreased with increasing *N*. The results observed for the grafted PMA chains were consistent with what has been observed when polymer chains tethered to rigid NP cores transitioned between a highly stretched to a more relaxed brush regime [21,45]. In addition, it was found that the glass transition temperature of PS core (Figure 4b; Table S1, Supplementary Material) significantly decreased by grafting PMA chains to the PS core. The grafting of the PMA chains on the surface of the PS core softened the core, in keeping with the increased local subsegmental mobility observed by NMR relaxation in the swollen nanoparticles suspensions.

## 4. Conclusions

In conclusion, using NMR relaxation, we showed that the local segmental dynamics of PS-PMA NPs in suspension depends on the architecture of the system. The degree of polymerization and the grafting density influenced the dynamics of the interfacial layer of end-tethered PMA chain, as observed in other systems where mobile chains were grafted to rigid substrates, but also influenced the dynamics of the PS core. The results presented here also show a unique phenomenon: the grafting of a polymer canopy to a swollen and deformable PS core also influenced the dynamics of the core. The grafting of PMA chain to the PS core facilitated the subsegmental relaxation of the PS network. The fastest local PS dynamics were observed when long chains were densely grafted to the PS core. The cooperative nature of the relaxation decreased for the grafted PMA chains in comparison to the free PMA chains. However, this effect was amplified for short chains, and this can be related to the conformational change in the polymer chains upon grafting. The local subsegmental motion of the PS-PMA NPs in suspensions correlates with the global thermal movement of the system in dry state measured by differential calorimetry.

## Figures and Tables

**Table 1:** Library of PS-PMA core-canopy NPs.

Sample	Grafting Density (Chains nm⁻^2^)	*N*	M_n, NMR_ (kDa)	M_n, SEC_ (kDa)	*Ð*	*R*_s_ in DCM (nm)
PS-σ_low_	0.07 ± 0.01	0				81 ± 20
PS-PMA_3k_-σ_low_		35	3	4	1.8	86 ± 20
PS-PMA_6k_-σ_low_		70	6	8	2.1	85 ± 30
PS-PMA_18k_-σ_low_		209	18	19	2.3	90 ± 20
PS-PMA_28k_-σ_low_		325	28	15	2.2	100 ± 30
PS-PMA_41k_-σ_low_		476	41	18	2.7	100 ± 20
PS-PMA_49k_-σ_low_		569	49	34	2.2	110 ± 20
PS-σ_medium_	0.46 ± 0.02	0				70 ± 20
PS-PMA_3k_-σ_medium_		30	3	4	1.9	90 ± 30
PS-PMA_7k_-σ_medium_		81	7	9	2.6	90 ± 30
PS-PMA_20k_-σ_medium_		232	20	20	2.2	120 ± 40
PS-PMA_30k_-σ_medium_		348	30	27	2.1	170 ± 30
PS-PMA_42k_-σ_medium_		488	42	29	2.1	160 ± 30
PS-PMA_51k_-σ_medium_		592	51	35	2.4	180 ± 40
PS-σ_high_	1.00 ± 0.12	0				90 ± 20
PS-PMA_3k_-σ_high_		35	3	4	1.7	110 ± 50
PS-PMA_10k_-σ_high_		116	10	12	2.1	130 ± 30
PS-PMA_20k_-σ_high_		232	20	21	2.1	160 ± 70
PS-PMA_33k_-σ_high_		383	33	27	2.4	190 ± 30
PS-PMA_42k_-σ_high_		488	42	40	2.5	200 ± 60
PS-PMA_55k_-σ_high_		639	55	44	2.0	240 ± 60

**Table 2:** Grafting density and scaling exponent of PS-PMA core-canopy NPs in DCM and H_2_O.

Grafting Density (σ)	Grafting Density in H_2_O (Chains nm⁻^2^)	Grafting Density in DCM (Chains nm⁻^2^)	Scaling Exponent in H_2_O	Scaling Exponent in DCM
Low	0.17	0.07	0.27	0.63
Medium	0.80	0.46	0.50	0.88
High	2.50	1.00	0.55	0.91

## Supplementary Materials

The following are available online at https://www.mdpi.com/2073-4360/12/8/1844/s1, Figure S1: Inversion-recovery NMR experiment, Figure S2: CPMG NMR experiment, Figure S3: Solvodynamic size distribution and TEM images of NPs, Figure S4: Temperature dependent *T*_2_ relaxation of PS-PMA NPs, Figure S5: Temperature and magnetic field strength dependent *T*_1_ relaxation of PS-PMA NPs, Figure S6: Temperature dependent swelling of NPs, Table S1: Glass transition temperature of PS and PMA.
