# Supplementary material for: Influence of the Architecture of Soft Polymer-Functionalized Polymer Nanoparticles on Their Dynamics in Suspension
Source: Polymers (Basel). 2020 Aug 17;12(8):1844. doi: 10.3390/polym12081844 (PMC7465671; doi:10.3390/polym12081844)
Supplement: Supplementary file 1 [file polymers-12-01844-s001.pdf]

Influence of the Architecture of Soft Polymer-Functionalized Polymer Nanoparticles on their Dynamics in Suspension

Young-Gon Kim, Waraporn Wichaita and Héloïse Thérien-Aubin *

Max Planck Institute for Polymer Research, Ackermannweg 10, 55131 Mainz, Germany; kimyoung@mpip-mainz.mpg.de (Y.-G.K); waraporn.wic@gmail.com (W.W.)

* Correspondence: therien@mpip-mainz.mpg.de

Supporting Information

1. NMR EXPERIMENTS

Measurement of the Spin-Lattice relaxation (T_1)

The PS-PMA core-canopy NPs were dispersed in CD_2Cl_2 at a concentration of 16.7 mg mL^{-1}. The relaxation experiments were performed on a series of NMR AVANCE spectrometers (Bruker) working at a nominal frequency of 500.13, 700.02 and 850.27 MHz. The spin-lattice relaxation time constants (T_1) were measured using a standard inversion-recovery pulse sequence (180–τ–90–acquire). The recovery time (τ) was varied from 20 ms to 10 s and the temperature was set at 278, 288, 298 and 308 K. The protons in the aromatic ring of PS (7.1 to 6.6 ppm) and in methoxy group (3.7 ppm) were chosen for the analysis. The area of the NMR peak at each recovery time was fitted with a monoexponential decay (equation S1) to calculate the relaxation time T_1.

$$\frac{I(\tau)}{I(0)} = A \times (1 - 2 \times \exp(\frac{-\tau}{T_1})) \quad \text{(S1)}$$

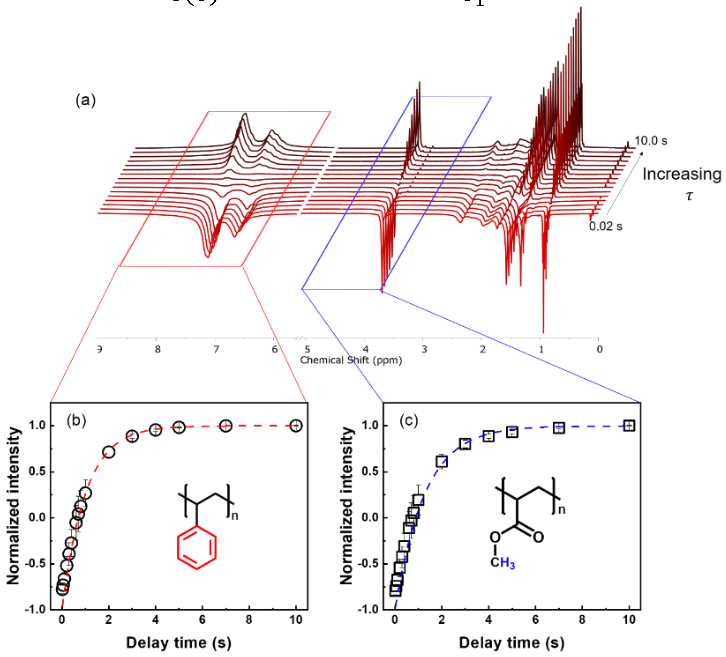

Figure S1 (**a**) Inversion-recovery NMR experiment of PS-PMA NPs at a Larmor frequency of 500.13 MHz at 298K. ^{1}H magnetization recovery for (**b**) aromatic protons of PS and (**c**) methoxy protons of PMA in PS-PMA NPs fitted with equation S1.

Measurement of the Spin-Spin relaxation (T_2)

The PS NPs, PS-PMA NPs and PMA free chain were dispersed in deuterated solvent mixtures at a concentration of 16.7 mg mL^{-1}. The relaxation experiments were performed on NMR AVANCE spectrometers (Bruker), working at a nominal frequency of 300.13 MHz. The spin-spin relaxation time constants (T_2) were measured using the Carr-Purcell-Meiboom-Gill (CPMG) pulse sequence using 16 spin-echo times (τ). The τ was varied from 4 ms to 8.3 s and the temperature was set at 298 K. The protons in the aromatic ring of PS (7.1 to 6.6 ppm) and in the methoxy group (3.7 ppm) were chosen for the analysis. The area of the NMR peak at each spin-echo time (M) was fitted with a stretched exponential function (equation S2) to calculate the apparent relaxation constant $T_{2,\,App}$. The average relaxation $T_{2,\,Ave}$ was obtained from the equation S3.

$$M(\tau) = A_0 \times \exp(-(\frac{\tau}{T_{2,App}})^{\beta}) \quad \text{(S2)}$$

$$T_{2,Ave} = \frac{T_{2,App}}{\beta} \times \Gamma(\frac{1}{\beta}) \quad \text{(S3)}$$

where β represents the width of the distribution and Γ is the gamma function.

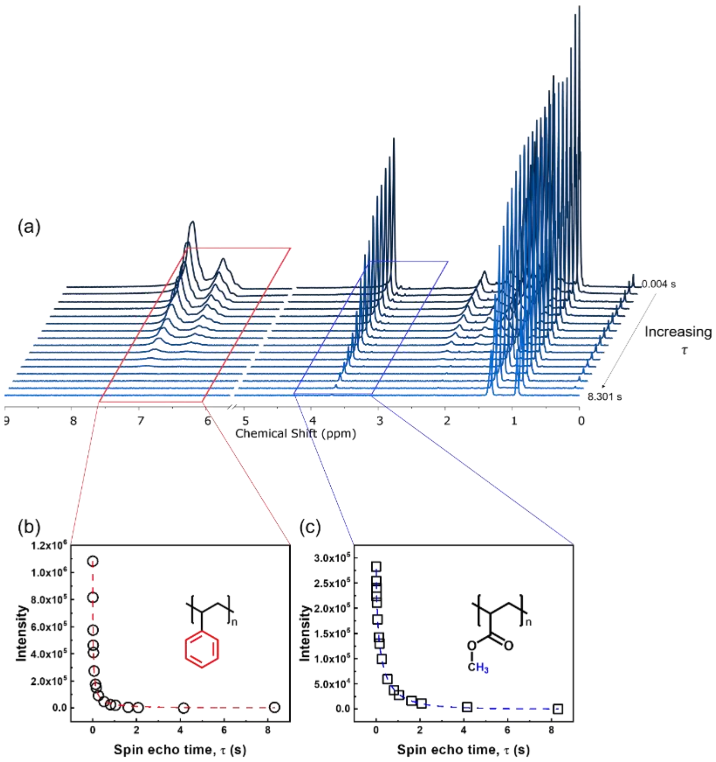

Figure S2. (**a**) CPMG NMR experiment of PS-PMA NPs at a Larmor frequency of 500.13 MHz at 298 K. ^{1}H magnetization recovery for (**b**) aromatic protons of PS and (**c**) methoxy protons of PMA in PS-PMA NPs fitted with equation S1

2. TEM MEASUREMENTS

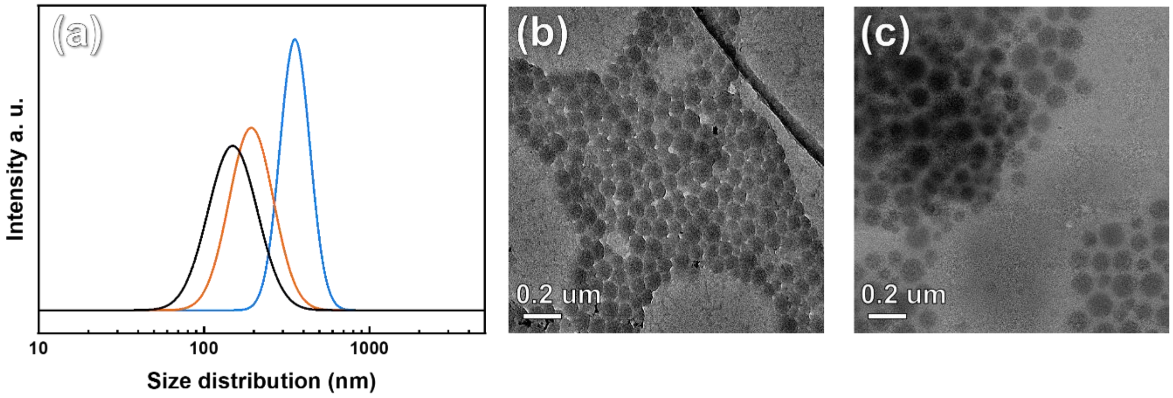

Figure S3. (**a**) Solvodynamic size distribution of PS NPs (**black line**), PS-PMA$_{3k}$ NPs (**orange line**) and PS-PMA$_{51k}$ NPs (**blue line**) with medium grafting density in DCM measured by DLS and transmission electron microscopy image (TEM) of (**b**) PS-PMA$_{3k}$ and (**c**) PS-PMA$_{51k}$ NPs with medium grafting density. TEM was analyzed by using an FEI Tecnai F20 operated with an accelerating voltage of 200 kV.

3. RELAXATION RESULTS

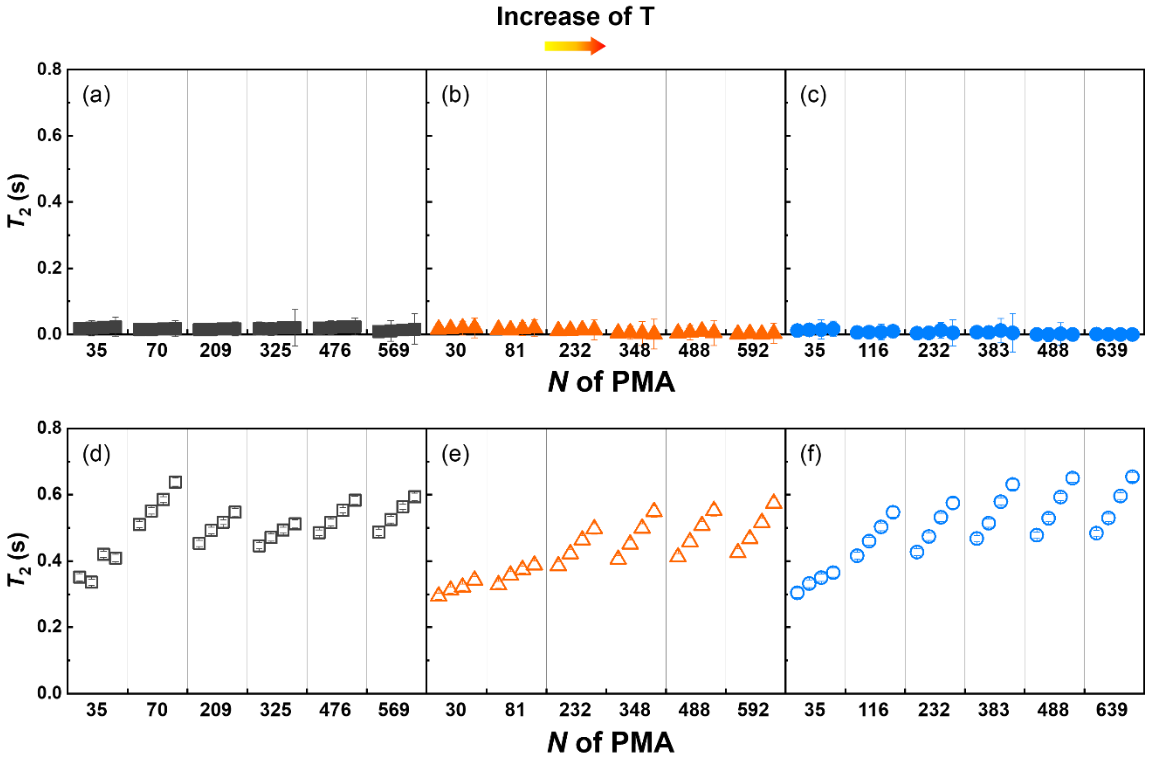

Figure S4. Temperature dependent ^{1}H spin–spin relaxation time of (a), (b), (c) aromatic ring of the PS core and (d), (e), (f) methoxy group of PMA canopy in the PS–PMA NPs with different degrees of polymerization of the PMA chains (*N*) grafted on PS cores with different grafting density, measured at 278K, 288K, 298K and 308K by using NMR spectrometer at a Larmor frequency of 700.02MHz. For PS-PMA NPs with low- (0.17 chains nm^{-2}, □), medium- (0.8 chains nm^{-2}, △) and high-grafting density (2.5 chains nm^{-2}, ○).

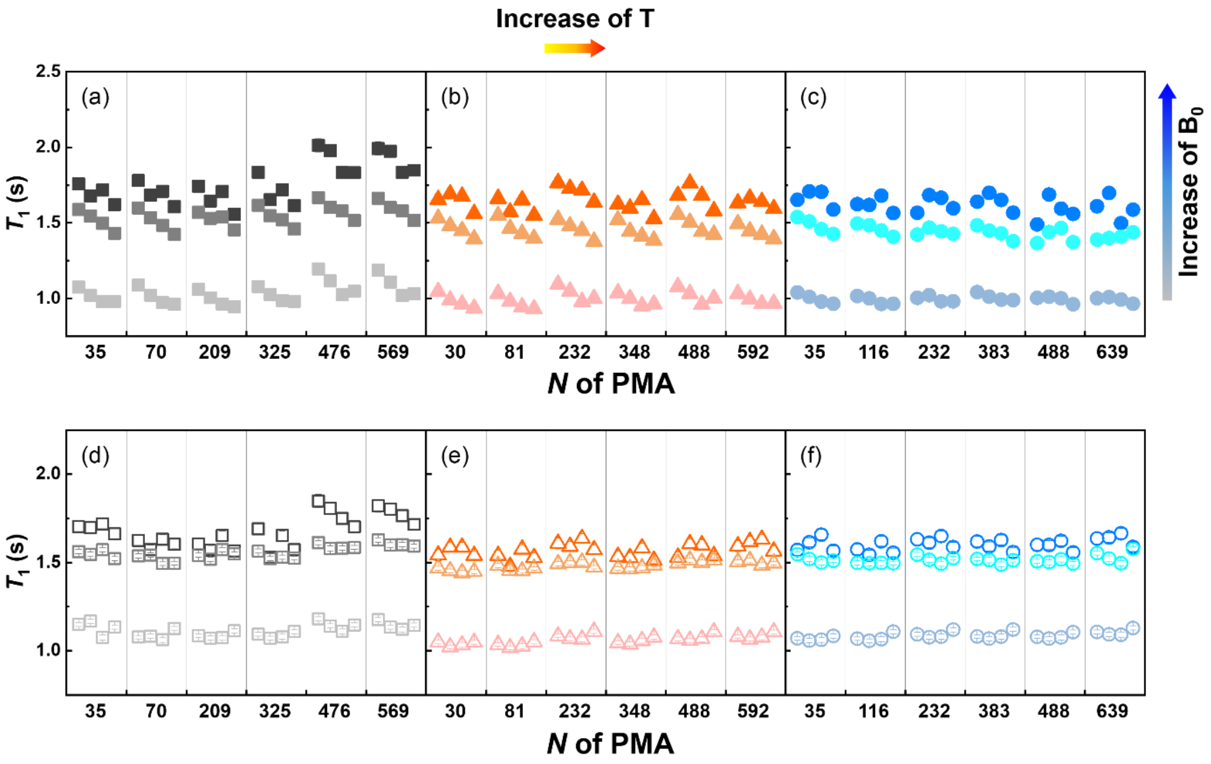

Figure S5. Temperature and magnetic field strength dependence of ^{1}H spin-lattice relaxation time of (a), (b), (c) PS core and (d), (e), (f) of PMA canopies in the PS-PMA NPs with low (0.17 chains nm^{-2}, □), medium (0.8 chains nm^{-2}, △) and high (2.5 chains nm^{-2}, ○) grafting density at Larmor frequencies of 500.13 MHz, 700.02 MHz and 850.27 MHz NMR at 278 K, 288 K, 298 K and 308 K. The magnetic field strength increases from the bottom to the top. The temperature rises from the left (278 K) to the right (308 K) within one column.

4. TEMPERATURE DEPENDENT SWELLING

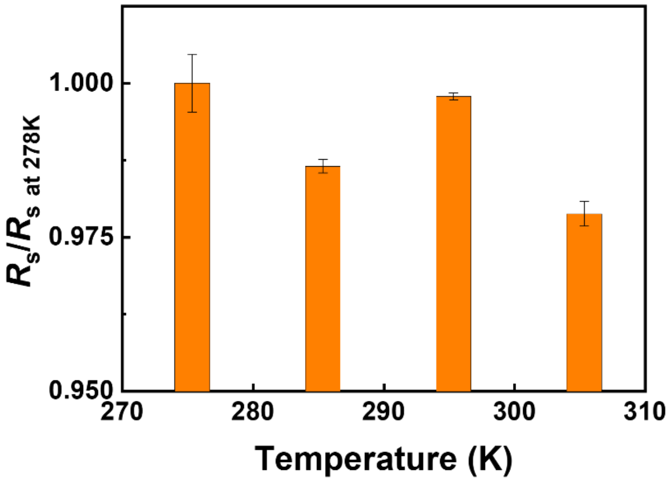

Figure S6. Normalized solvodynamic radius of pure PS NPs in DCM at given temperatures normalized by solvodynamic radius of NPs at 278K.

5. THERMAL ANALYSIS

Table S1 Glass transition temperature of PS and PMA, measured by DSC.

**Type of samples**	***N* of PMA**	**T_g of PMA (°C)**	**T_g of PS (°C)**
PS-PMA with Low σ	0		107.7
	35		98.4
	70		99.2
	209		100.9
	325		99.7
	569	14.4	
PS-PMA with Intermediate σ	0		107.9
	81	17.2	
	232	13.4	
	348	17.0	
	488	16.0	
	592	13.7	
PS-PMA with High σ	0		108.3
	35		99.0
	116	16.2	
	232	18.9	
	383	13.0	
	488	13.8	
	639	13.8	
Free PMA chain	46	11.8	
	116	10.3	
	186	10.9	
	279	12.2	
	523	13.9	
	616	13.8	
